# Petroleum Hydrocarbon Fingerprints of Water and Sediment Samples of Buffalo River Estuary in the Eastern Cape Province, South Africa

**DOI:** 10.1155/2017/2629365

**Published:** 2017-05-30

**Authors:** A. O. Adeniji, O. O. Okoh, A. I. Okoh

**Affiliations:** ^1^SAMRC Microbial Water Quality Monitoring Centre, University of Fort Hare, Alice 5700, South Africa; ^2^Department of Chemistry, University of Fort Hare, Alice 5700, South Africa; ^3^Applied and Environmental Microbiology Research Group, Department of Biochemistry and Microbiology, University of Fort Hare, Alice 5700, South Africa

## Abstract

Petroleum hydrocarbon status of the Buffalo River Estuary in East London, South Africa, was evaluated from January to May, 2016. Surface water and sediment samples were collected from five points in the estuary and extracted using standard methods. The extracts were subsequently analyzed by gas chromatography-flame ionization detection. Results showed that total petroleum hydrocarbon (TPH) varied from 7.65 to 477 *μ*g/L in the water and 12.59 to 1,100 mg/kg in the sediments, with mean values of 146.50 ± 27.96 *μ*g/L and 209.81 ± 63.82 mg/kg, respectively. Concentrations of TPH in the sediments correlated significantly with organic carbon (OC) in both seasons. TPH and OC levels were slightly lower in summer than in autumn in the two environmental matrices, and the average amount of TPH in the water samples collected from all the sampling stations was generally lower than the EU standard limit of 300 *μ*g/L. However, the levels in the sediments exceeded the EGASPIN target value (50 mg/kg) for mineral oil but were below the intervention value (5,000 mg/kg), indicating a serious impact of industrial growth and urbanization on the area, although the n-alkane ratios and indexes used for source tracking revealed excessive flow from both natural and anthropogenic sources.

## 1. Introduction

The presence of chemical contaminants in the coastal environments from many anthropogenic sources is a major threat to the marine water [1, 2]. Large amounts of these contaminants through sewage, petroleum spills, municipal and industrial discharges, and automobile wastes and vehicular emission due to incomplete combustion of fossil fuels are possibly carried by river runoffs through their estuaries into the sea [3, 4]. Petroleum hydrocarbons are one of the major pollutants which are frequently discharged into the coastal water, though not usually regulated as hazardous wastes [5, 6]. Although they are naturally present in very low concentration in the marine sediments, larger amount comes from petrogenic and pyrogenic sources [7–9] and bottom sediment which is the habitat of many aquatic organisms is recognized as a potential reservoir of the petroleum hydrocarbons in the marine environments, posing risk of bioaccumulation [4, 6].

Assessment of the physicochemical properties of the water and sediment is also very important because these quality parameters are capable of affecting the biological characteristics of the environmental matrices and are the basis for judging the suitability of such water body for its designated uses [10–12]. Although some of these parameters may have limited health significance, international standards necessitate their determination [13]. The physicochemical characteristics of each n-alkane in water and sediment samples will therefore depend on the source of contamination in the environmental matrices [14].

Some studies recently conducted on the physicochemical parameters of different water bodies across the globe were reported. Among the qualities assessed are pH, dissolved oxygen, temperature, turbidity, conductivity, total dissolved solids, and total suspended solids [11, 15, 16]. Analysis of sediments qualities has also been found very paramount being a major site for organic matter decomposition. Continuous accumulation of pollutants due to biological and geochemical mechanisms can be toxic to the sediment dwelling organisms and fish, resulting in decreased survival, reduced growth, or impaired reproduction and lowered species diversity. Some recent findings on quality parameters of sediment like pH, electrical conductivity, moisture content, organic carbon, and organic matter were also reported [17, 18].

Buffalo River Estuary is the mouth part of the long Buffalo River that covers about 1287 km^2^ area before draining into the Indian Ocean in the coastal city of East London. The river flows through some major towns (Bhisho, King William's Town, Zwelitsha, Mdantsane, and East London) and industrial areas that cover almost 12% of its catchment area. Three major tributaries that feed the Buffalo River with runoffs, raw sewage, solid wastes, and industrial effluents are the Mgqakwebe, Ngqokweni, and Yellowwoods rivers mainly from densely populated urban and agricultural areas of the province [19]. Surrounding the estuary are the East London harbour and other direct users like Sea Spirit Fish Market, Vukani Petroleum, BP South Africa, Mercedes Benz South Africa, Chevron (Pty) Ltd., Engen, and Total South Africa among others. First and Second Creeks are instances of influent rivers which contribute to the inflow of stormwater, sewerage, and domestic and industrial runoffs into the estuary [20].

Previous environmental studies in the area focused on the physicochemical characteristics and microbial and heavy metal contamination of the environmental matrices [19]. However, no report is available yet on the petroleum hydrocarbon profiles in both the water and sediment compartments of the estuary, which is the subject of this current study. In this paper, we report on the petroleum hydrocarbon fingerprints of water and bottom sediments of the Buffalo River Estuary in the Eastern Cape Province, South Africa.

## 2. Materials and Method

### 2.1. Description of Study Area

The Buffalo River Estuary (33°02′S; 27°55′E) is the largest and one of the most important river estuaries in the Eastern Cape Province of South Africa and located in the Buffalo City Metropolitan Municipality, East London [19]. It receives stormwater from the shipping activities at the East London harbour, industrial effluents, domestic wastes, urban runoff, and vehicular emissions from Steve Biko and Buffalo Bridges either directly or indirectly through its influent rivers.

### 2.2. Cleaning of Glass Bottles and Sample Collection

Amber glass bottles used for sample collection were washed with tap water and detergent and rinsed with distilled water followed by acetone. Calibration standards, high performance liquid chromatographic (HPLC) grade solvents, and analytical grade reagents used for this work were sourced from Merck (Germany) and Restek and Accustandard (USA).

**Figure 1:**
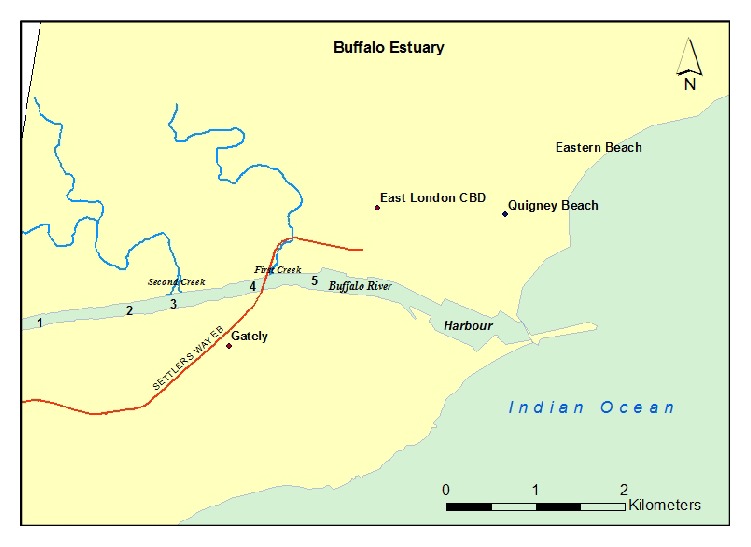
Map of Buffalo River Estuary, East London.

Five (5) sampling locations on the Buffalo River Estuary were chosen on the basis of the level of anthropogenic pressures (Table 1; Figure 1). Monthly sampling was undertaken between January and May, 2016, mostly during the high tide except in February, spanning through summer and autumn seasons. Duplicate water samples were collected 100 mm below the surface level from two points (about 5 m apart) in each sampling location with precleaned 1 L amber bottles, pooled together to obtain a representative sample [21]. They were acidified to pH < 2 using 6 M hydrochloric acid (HCl). The sediment samples were however collected only from 4 locations using Van Veen Grab sampler, because the estuary substratum at E5 was rocky. All the samples were transported to the laboratory immediately on ice chest for analysis [22, 23].

### 2.3. Physicochemical Analyses of the Samples

Temperature, pH, electrical conductivity (EC), and total dissolved solids (TDS) of the aqueous samples were determined* on-site* using Hanna multiparameter probe (HI 98195), while turbidity, dissolved oxygen, and total suspended solids (TSS) were measured using Hach instruments (Hach Company, USA) [24]. Sediment samples were analyzed for moisture, organic carbon, and organic matter contents using gravimetric method [25, 26].

### 2.4. Extraction of Petroleum Hydrocarbon from Surface Water and Sediment Samples

Exactly 500 mL of water sample was spiked with 1 mL of 10 *μ*g/mL 1-chlorooctadecane (COD) used as surrogate standard, extracted thrice in a separating funnel with 20 mL of n-hexane each time. The extracts were pooled together, dried with anhydrous sodium sulphate (20 g), and concentrated to about 2 mL using rotary evaporator, ready for column cleanup [25, 27, 28]. Sediment samples were air-dried for 5 days. Also, 10 g aliquot of the air-dried sediment sample was blended with sufficient quantity of anhydrous sodium sulphate (Na_2_SO_4_) to remove moisture, spiked with 1 mL of 10 *μ*g/mL COD and extracted in a Soxhlet extractor with 200 mL of dichloromethane for 24 h. The extract was allowed to pass through a glass funnel containing anhydrous sodium sulphate and reduced to approximately 2 mL using rotary evaporator. It was then solvent exchanged to n-hexane before the cleanup stage [25, 29].

### 2.5. Silica Gel Cleanup and Separation

The water and sediment extracts were transferred into a 10 mm ID × 30 cm chromatographic column packed with 10 g activated silica gel slurry with about 2 cm anhydrous sulphate layer on top. The column was eluted with 20 mL of n-pentane to obtain the aliphatic hydrocarbon fraction [30]. The eluates were concentrated to about 2 mL with rotary evaporator and solvent exchanged to n-hexane. A control (blank) sample was treated the same way as the real sample for quality assurance [25, 31].

### 2.6. Gas Chromatography Analysis

All the cleaned sample extracts were analyzed using Agilent 7820A gas chromatograph (GC) equipped with flame ionization detector and HP-5 fused silica capillary column (30 m × 0.32 mm ID × 0.25 *μ*m film thickness). The carrier gas was helium at flow rate of 1.75 mL/min and average velocity of 29.47 cm/sec. Exactly 1 *μ*L of the sample extract was injected in splitless mode at 300°C. The column temperature was held at 40°C for 1 min and then increased at 7°C/min to 320°C. The detector temperature was 300°C [1, 28].

The gas chromatograph was calibrated with n-alkane working standards prepared in the range of 0.05–20 *μ*g/mL using n-hexane as diluent. Calibration curves were plotted and average response factor was generated with Agilent Chemstation chromatography software for each analyte. The curves were linear with correlation coefficients ranging from 0.9846 to 0.9919. The unresolved peaks were quantified using the response factor of nC15 in accordance with the method of Luan and Szelewski [32]. TPH was integrated with baseline-holding and peak sum slicing and then quantified as the sum of concentrations of the n-alkanes that eluted from nC9 to nC36 and UCM. Data analysis was done with the Agilent software [14, 32] while ANOVA and Pearson correlation were performed using IBM SPSS Statistics 20, with the level of significance set at 0.01 [11].

Limits of detection (LOD) for n-alkanes were determined from 8 replicate injections of a middle level calibration standard [33, 34]. LOD was statistically calculated by multiplying the standard deviation of the instrument response by “*t*” value at 99% confidence level [35].

## 3. Results and Discussion

### 3.1. Physicochemical Properties of Buffalo River Estuary Water Medium

Table 2 shows the seasonal variation and the range of values obtained for the physicochemical analyses of water from the five sampling locations in this study while Table 3 demonstrates the Pearson correlation among the water quality parameters.

pH is an important indicator of water quality and pollution level in the aquatic environment. It is closely linked with biological productivity [36]. The pH of the water samples collected in the study area ranged between 7.4 and 9.3. The values recorded in summer were generally higher than autumn. However, there was no significant difference in the pH values across the 5 sampling stations. The South African permissible limit for coastal and marine water is 5.0–8.0 [37] and the EU target limit is 6.0–9.0 [38]. The pH values recorded in this study were within the stipulated limits, except in January in four of the five stations (9.1–9.3). A significant positive correlation was observed between pH and other parameters including temperature (*r* = 0.888; *p* < 0.01), conductivity (*r* = 0.560; *p* < 0.01), and salinity (*r* = 0.861; *p* < 0.01) (Table 3). Reportedly, higher pH values have been linked to high concentration of carbonate in the water body [16, 35].

Water temperature is another priority quality indicator that plays a vital role in aquatic systems due to its ability to cause mortality and as well influence the solubility of dissolved oxygen needed by the aquatic lives [36]. In the current investigations, temperature varied from 17 to 25°C in the water column. Temperature was generally higher in summer than in autumn, though with no statistical difference among the sampling stations, although all the values obtained throughout the study period fell within the South African threshold limit of 15–35°C [33]. Temperature also correlates positively with salinity (*r* = 0.864; *p* < 0.01) and TDS (*r* = 0.391; *p* < 0.05) as earlier reported but no correlation with dissolved oxygen was observed [39].

Conductivity is defined as a measure of the ability of a solution to conduct electrical current through the water [24]. It is usually higher in saline compared to freshwater systems [36]. The conductivity of the estuary water varied from 24.20 to 40.17 *μ*S/m (Table 2). It was generally lower in summer (mean = 27.76 *μ*S/m) than in autumn (mean = 34.47 *μ*S/m) and showed a positive relationship with depth, increasing as the depth increases. A similar trend was observed in the salinity. There is no standard guideline set for the control of conductivity in marine water, although limits set for total dissolved solids are sometimes used in its stead [40]. Effluent or sewage discharge, as well as dissolved ions from soil and rocks, are known to influence the values of conductivity and salinity in the water column [41, 42]. Significant correlation (*r* = 0.670; *p* < 0.01) observed between conductivity and salinity as shown in Table 3 confirmed their positive relationship. pH also correlates positively with conductivity (*r* = 0.560; *p* < 0.01) as previously reported [39].

Total dissolved solids (TDS) value is a measure of inorganic and organic materials present in water, of which salt forms the principal constituent [42]. The TDS of the estuary water ranged between 13.50 mg/L and 22.75 mg/L. The trend was similar to that of the conductivity where the values were higher in autumn than in summer. Although South Africa and EU do not have any target value for TDS in the coastal and marine water, the results obtained were higher than 10 mg/L set as target value for estuary and coastal water in the Darwin Harbour region [43]. TDS in this work correlate positively with salinity (*r* = 0.717; *p* < 0.01) in agreement with the report of Chigor et al. [11], conductivity (*r* = 0.835; *p* < 0.01), pH (*r* = 0.451; *p* < 0.05), temperature (*r* = 0.391; *p* < 0.05), and dissolved oxygen (*r* = 0.406; *p* < 0.05) as shown in Table 3. A general increase in concentration was observed as the water depth increases [40]. Waters with high TDS cause cells to shrink, disrupt organisms' movement, and make them afloat or sink beyond their normal range. High TDS value indicates high alkalinity or hardness and can consequently affect taste of the water column [40].

Dissolved oxygen (DO) is another important component of aquatic system [16, 36, 41]. It is a measure of the level of free, noncompound (nonbonded) oxygen dissolved in the water column [44]. DO enrichment in surface water is permitted through atmospheric exchange. However, thermal or salinity stratification and anthropogenic activities involving organic matters may result in DO depletion, suppressing respiration and causing huge die-offs of recreationally indispensable fish [39, 45]. The results of the DO measured in the estuary water ranged as 2.41–9.19 mg/L (Table 2). The least average DO was observed in summer when the water temperature was the highest, supporting an inverse relationship between the two parameters as evidently revealed (Table 3) [36]. Although there was no significant difference spotted between the stations, however a gradual increase was observed from summer to autumn and also as depth increases. The minimum requirement of dissolved oxygen in coastal water is 4 mg/L [39]. DO concentrations recorded in this study area were higher than the standard limit in all the sites except only once in summer when a lower value of 2.41 mg/L was obtained at site E2 [46].

Turbidity and total suspended solids (TSS) are other quality parameters with significant relationship. Turbidity is a measure of water clarity or muddiness resulting from the collection of dissolved and suspended solids, making light scattered and absorbed instead of being transmitted in straight lines [47]. Elevated turbidity in waters is often linked with the likelihood of microbiological pollution [41]. The current investigation had mean turbidity of 47.86 ± 19.58 NTU for the estuary water in the range of 22.9–100 mg/L. Turbidity was generally higher in autumn than in summer. The highest value of 100 mg/L was obtained at E3 (second creek) in May when fresh industrial effluents were discharged into the estuary and the lowest was also recorded the same month at E5 (22.9 mg/L). There was a gradual decrease in turbidity values as the depth increases and from summer to autumn. Turbidity recorded at the shallowest site (E1) was significantly different from the values obtained from the two deepest sites, E4 (*p* < 0.01) and E5 (*p* < 0.05).

Similarly, significantly high TSS was recorded in May at E3 (447 mg/L). This could be attributed to the fresh discharge of effluents sighted at the site during the time of sampling. Total suspended solids in the estuary generally ranged from 1.33 mg/L to 447 mg/L showing the same trend of decrease as the water depth increases. There was no significant difference in TSS among the five (5) sampling points. Higher turbidity in the nearshore waters may be due to particulates arising from clay and silts from shoreline erosion, resuspended bottom sediment, organic detritus from water discharges, urban runoff, industrial effluents, and excess phytoplankton growth [24, 47].

### 3.2. Levels of n-Alkanes and Total Petroleum Hydrocarbons in Water and Sediment Samples

#### 3.2.1. Quality Control

The quality control study was carried out by spiking surrogate standard into each water and sediment sample before extraction and the recoveries which were within the acceptable range of 40–140% were used for correcting the final concentrations of the analyte compounds extracted. Limit of detection obtained for the n-alkanes varied from 0.06 to 0.13 *μ*g/L and the relative standard deviations (RSD) were in the range of 3.61 to 8.32% [48, 49].

#### 3.2.2. Levels of Petroleum Hydrocarbons in the Water Samples

**Figure 2:**
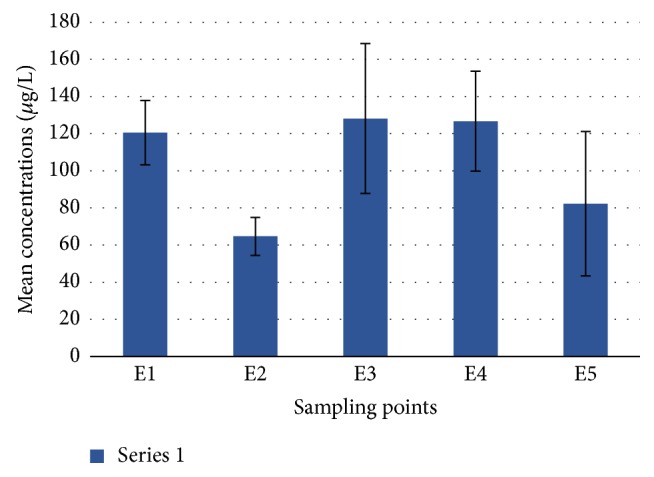
Spatial variation of TPH in the Buffalo River water samples.

Total petroleum hydrocarbons (TPH) in the surface water of Buffalo River Estuary were evaluated in all the sampling points. The results covering two seasons (summer and autumn) are summarized in Table 4 and spatial distribution of petroleum hydrocarbons in the water matrix is depicted in Figure 2 below.

The TPH concentration in the study varied widely across the 5 locations from 7.65 to 477.07 *μ*g/L. The highest concentration was observed at site E3, followed by sites E4 and E1 as shown in Figure 2. Among these, sites E3 and E4 which were the second and first creeks receive sewerage, stormwater, and runoff from industrial (including Gately, West Bank Hood, Woodbrook, and East London Harbour) as well as the residential and commercial areas in the East London metropolis. Site E1 was located at the entry point of the estuary where Buffalo River freshwater is discharged with pollution loads from some major towns like King Williams Town, Zwelisha, and Mdantsane. Lowest concentrations of TPH however occurred in sites E5 and E2 with seemingly less anthropogenic activities [19, 20]. Depth may also have contributed to the low concentration of TPH in site E5 [50].

No particular trend was observed in the distribution of TPH across the whole period of study. Generally, the concentrations were lower in summer than in autumn as reported by Maktoof et al. [51]. This could be linked with the collection of samples mostly during the high tide in autumn compared to summer [52] and also because larger amount of pollutants are swept into water bodies in autumn as the level of rainfall increases [53]. The highest concentration which was recorded at site E3 in May was probably due to the fresh discharge of effluent at the time of sampling. Statistical analyses however did not show any significant difference in the TPH concentrations obtained across the stations. The total mean concentration of TPH (146.50 ± 27.96 *μ*g/L) in all the sites was lower than the EU acceptable standard limit for hydrocarbons (300 *μ*g/L) in estuary and harbour basin water [38].

The level of water TPH from this study was significantly lower compared to values reported in some regions including Bohai Bay of China [54], Strait of Johor, Peninsular Malaysia [55], seawater of North Cape [56], groundwater samples collected from some communities in Rivers State, Nigeria [57], surface water from Ubeji in Warri, Nigeria [58], and the surface water from the neighbourhood of Nigerian National Petroleum Corporations Oil Depot in Apata, Ibadan Metropolis, Nigeria [59]. However, some studies were reported with lower values of TPH than obtained in this study in other parts of the world. Examples include water samples from Main Outfall Drain in Al-Nassiriya City/Southern Iraq [51], water samples from Setiu Wetland [60], Terengganu coastal waters from Malaysian west coast [55], and Dungun River basin water, Malaysia [23].

#### 3.2.3. Levels of Petroleum Hydrocarbons in the Sediment Samples

**Figure 3:**
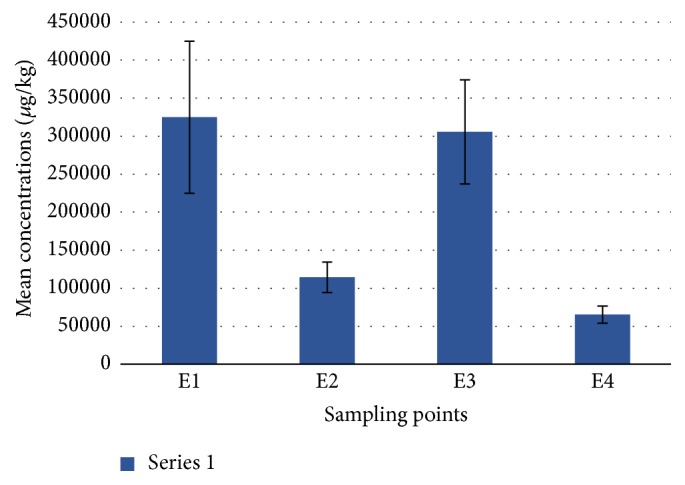
Spatial variation of TPH in the Buffalo River sediment samples.

In this study, the concentrations of TPH in the estuary sediments expressed on dry weight basis are presented in Table 5. The values ranged from 12.59 to 1,100 mg/kg. As shown in Figure 3, site E1 recorded the highest concentration of TPH, followed by site E3 (second creek) while the lowest was obtained from site E4, which could possibly be related to the higher depth of water at the station [50]. However, the rocky substratum of site E5 made sediment sampling difficult throughout the span of this study. There was no doubt that larger contribution of pollutants in the estuary sediment was from Buffalo River water discharge at E1, as well as the introduction of the industrial effluents and leachates from the old land fill site at E3 among others [19, 20]. Notwithstanding, the statistical tests conducted revealed no significant difference in the levels of TPH determined between the sampling stations. The temporal distribution of TPH in the sediment follows this order: April > January > May > March > February (Table 5). Petroleum hydrocarbons in the sediments were found much higher in autumn with mean concentration of 233.62 mg/kg than in summer (160.22 mg/kg) due to the upward push and resuspension of contaminants occasioned in some cases by the high tidal forces in the aquatic environment [52, 61].

Although no sediment guideline is in place for total petroleum hydrocarbons, nevertheless, four levels of petroleum hydrocarbon pollution were suggested for the assessment of marine sediments [50]. They were unpolluted (10–15 mg/kg), slightly polluted (15–50 mg/kg), moderately polluted (50–200 mg/kg), and heavily polluted statuses (>200 mg/kg). This was not entirely different from the respective target and intervention values of 50 mg/kg and 5,000 mg/kg set as environmental guidelines for soil and sediment TPH by the Department of Petroleum Resources, Nigeria [62, 63]. In light of the above, the TPH concentrations of the Buffalo River Estuary sediments were largely below the intervention value, ranging from “moderately polluted” in summer to “heavily polluted” in autumn [50, 64].

Petroleum hydrocarbon levels in the sediment compartment of this study area were found comparatively lower than those reported for the sediments from Ceuta harbour, North Africa [65], and Arabian Gulf, Kuwait [50]. However, the concentrations were a little higher than those from the Arabian Gulf [66], Bohai Bay, China [67], Khowr-e Musa Bay [22], and Musa Bay [68]. Nonpolluted sediments reported in some studies include those from coastal area of Putatan and Papar, Sabah [69], Main Outfall Drain in Al-Nassiriya City, Southern Iraq [51], and coastline and mangroves of the northern Persian Gulf [70].

### 3.3. TPH Relationship with Percentage Moisture, Organic Carbon, and Organic Matter Contents of the Sediments

Table 6 shows the percentage moisture, organic carbon (OC), and organic matter (OM) of the estuary sediments. The muddy sediment samples contain a large percentage of water in the range of 45.01–67.86%, with an overall average of 59.03%. The sediment grains were relatively small, having an average composition of 58.35% fine sand, 30.43% very fine sand, and 11.22% coarse silt determined by sieving. Both OC and OM in muddy sediments were considered to play a significant role in the accumulation and release of various micropollutants in the aquatic environments [50].

**Figure 4:**
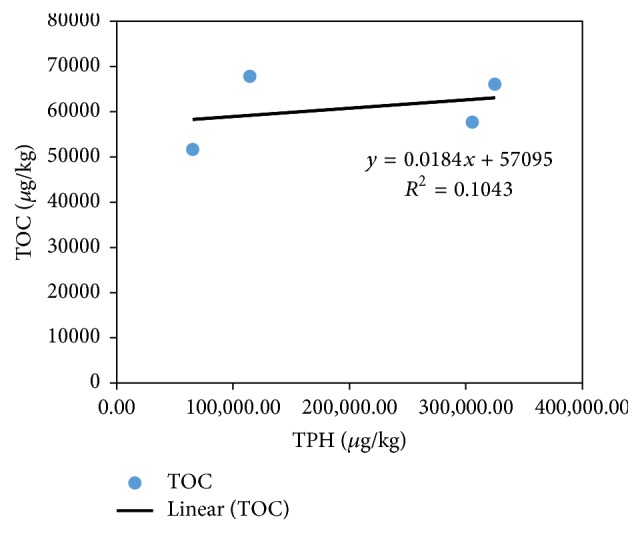
Plot of TOC versus TPH in the water matrix of Buffalo River Estuary.

Organic carbon in the sediment samples ranged from 3.12 to 8.94% (mean = 6.14%), whereas organic matter varied between 5.39% and 15.42% (mean = 10.59%). Statistical analysis showed a significant difference (*p* < 0.1) in the moisture contents of the sediments collected from E2 and E4. However, no significant difference was observed among the stations in relation to OC and OM. The total petroleum hydrocarbon positively correlate with OC (*r* = 0.665; *p* < 0.01) and OM (*r* = 0.665; *p* < 0.01) (Table 7), indicating a direct relationship among the three parameters. This relationship was further checked in a plot of OC against TPH. The plot yielded a straight line with *r*^2^ = 0.1043 (Figure 4). High concentration of petroleum hydrocarbons in the sediments of the river estuary testifies to the role of sediment as a sink for organic micropollutants [71]. Petroleum hydrocarbons concentration is usually higher in muddy sediments compared to the coarse ones, establishing a strong relationship between grain size and TPH [50, 72, 73].

### 3.4. Hydrocarbon Source Identification Using Molecular Markers

The use of n-alkanes as a molecular marker has been of great advantage in the identification of pollution sources in the estuarine and marine environments [9]. Many mathematical ratios and indexes like major hydrocarbon (MH), ratios of low molecular weight to high molecular weight* n-*alkanes (L/H), unresolved complex mixture (UCM), average carbon chain (ACL), carbon preference index (CPI), weathering index, and C31/C19 have been used to identify the origins of n-alkane in the environment [74, 75].

#### 3.4.1. Distribution of n-Alkanes

Various ways through which hydrocarbons come to the surface of marine and coastal waters include anthropogenic activities, biosynthesis (production of hydrocarbons by some living organisms in the aquatic environments), and geochemical processes (e.g., seepage). Some anthropogenic activities involving industries, oil operations, and urban undertakings could generate substantial quantity of hydrocarbons in the marine waters. However, hydrocarbons from biosynthetic marine origins are always present in trace quantity [9, 74].

Concentrations of total *n*-alkanes in the estuary vary between 15.46 and 328.59 *μ*g/L in the water samples and from 9,889 to 169,885 *μ*g/kg in the sediment samples as shown in Tables 4 and 5. Clear indication of different sources is offered by the strong presence of some specific normal alkanes. Examples include nC25–nC35 which indicate biogenic input (terrestrial and vascular plants), and a strong dominance of odd over even numbered n-alkanes could also suggest the same origin. The presence of nC15, nC17, and nC19 n-alkanes indicates inputs from phytoplankton and algae marine biogenic sources. Dominance of even carbon n-alkanes such as C16, C18, and C20 indicate petroleum hydrocarbons pollution from anthropogenic sources. Likewise, the presence of nC12 and nC14 alkanes indicates microbial biogenic origin [27, 76, 77].

Distribution of n-alkanes in the study revealed that the most prominent ones in the water samples were nC9–nC12, nC15, nC17, nC19, and nC26–nC36 whereas, in the sediment samples, nC10–nC12, nC18, nC20, nC22, and C22–nC36 dominate. Generally, heavier hydrocarbons were more in abundance than the lighter ones and odd carbon n-alkanes seemed to be a little more dominant than the even carbon n-alkanes as was also reported by Ahmed et al. [28]. Aliphatic hydrocarbons in the water compartment of the estuary are principally from biogenic sources. However, the origins of the n-alkanes in the sediments are both biogenic and anthropogenic (possibly from fishing, industrial storm water, and urban runoff) [78].

#### 3.4.2. Low Molecular Weight/High Molecular Weight n-Alkanes (L/H)

Ratio of low molecular weight n-alkanes (C15–C20) to high molecular weight *n-*alkanes (C21–C34) is another marker being used for the determination of *n*-alkanes sources [28, 78]. Low molecular weight hydrocarbon usually dominates in a fresh oil release, giving a ratio greater than one. L/H is an indicator of the freshness of the hydrocarbons released into the environments. However, fast degradation of these lower molecules in crude oil can decrease the ratio significantly to a value below unity. The L/H ratio below 1 (unity) reveals natural input from marine and terrestrial biogenic sources, and ratios around and above 1 indicate hydrocarbons from petroleum sources [79, 80].

The results obtained from the water samples showed the L/H ratios greater than one (1) in the first two stations (E1 and E2), indicating fresh release of petroleum hydrocarbons from agricultural, residential, and/or industrial activities that entered the estuary through rivers and streams in the watershed. However, the remaining three sampling locations yielded ratios lower than one (unity). Sediments samples from all the stations also gave L/H ratios lower than 1, suggesting hydrocarbons from natural sources [79].

#### 3.4.3. Long Chain Hydrocarbons/Short Chain Hydrocarbons (LHC/SHC)

Short chain hydrocarbons (SHC) are the n-alkanes which are below and up to nC26 (e.g., nC15, nC17, and nC19). They are derived from planktonic and benthic algal sources, whereas long chain hydrocarbons (LHC) are the n-alkanes up to nC26 (e.g., nC27, nC29, and nC31) which are mostly from vascular plants sources. They are known to be more resistant to decomposition than their short chain counterparts. The ratio is used to evaluate the dominance of vascular plant and phytoplanktons in the marine environments. While the ratio of LHC/SHC between 0.21 and 0.80 indicates phytoplankton sources, value in the range of 2.38 to 4.33 suggests a mixture of both sources. Higher value greater than 4.0 shows the dominance of terrestrial plant waxes [78, 81].

  LHC = sum of nC27, nC29, and nC31.
  SHC = sum of nC15, nC17, and nC19.

Average LHC/SHC ratio obtained in this study ranged between 11.18 and 17.71 in the 4 sediment stations, an indication of a significant input from terrestrial plant waxes.

#### 3.4.4. Unresolved Complex Mixture (UCM) and Weathering Index (WI)

The UCM is considered a mixture of branched and cyclic hydrocarbon structures, including many of their structurally complex isomers which are unresolvable by the capillary columns of gas chromatograph [74, 80]. The aliphatic hydrocarbons are less soluble than the aromatic compounds which are relatively more mobile in water. The branched aliphatics are even less water-soluble than the straight-chained alkanes. Hence, they concentrate more in the sediment compared to the water column [5]. Although UCM are mostly present in the higher molecular range of the hydrocarbons, few exist in the lower range as well. The lower range UCM can easily be evaporated unlike their counterparts in the higher range category and their presence in the marine environments generally indicate bacterial degradation of old petroleum products or chronic oil pollution; however fingerprinting information available from their data is very limited [9, 14, 82].

**Figure 5:**
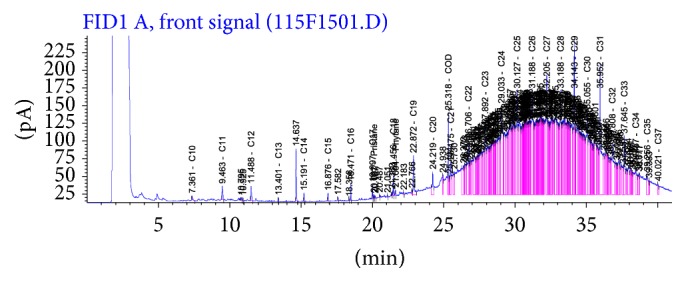
Chromatogram of a typical sediment sample from E1.

**Figure 6:**
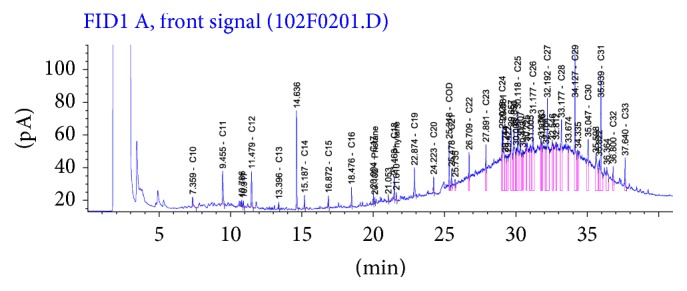
Chromatogram of a typical sediment sample from E4.

Figures 5 and 6 revealed the presence of UCM in some of the samples analyzed (mostly sediments) appearing as a unimodal or sometimes as a bimodal hump in the range of nC12 to nC15 and nC22 to nC35. The UCM concentrations in the sediments varied between 2,700 and 930,328 *μ*g/kg (mean = 160,278 ± 54.19 *μ*g/kg) and in the water samples between 67 and 279 *μ*g/L (mean = 66.66 ± 16.78 *μ*g/L). Meanwhile, the abundance of the higher molecular weight hydrocarbons (>nC23) over the lighter ones suggests no recent contamination in the aquatic environment [14, 83].

Another useful tool employed for the assessment of the presence of some important crude oil residues is the ratio of the unresolved components to that of the resolved ones (U/R), otherwise called weathering index. Higher ratio greater than 4 is usually a criterion for important petroleum residues [84]. In this study, U/R in the sediment samples generally ranged from 0.27 to 5.77. Higher ratios greater than 4 were observed only in autumn (March and April) at stations E1 and E3, suggesting pollution arising from the inflow of urban and industrial runoffs, effluent discharge, leachate from a nearby dumpsite, and stormwater from the nearby harbour where shipping activities are ongoing among other possible sources. However, the maximum value recorded in the water samples was 1.41, indicating a relatively low petroleum contamination in the water column. Degraded petroleum residues may stay bound to soils/sediments for years and may bring about a short-term damage to aquatic lives and also impact the recreational use of the water body negatively [5].

#### 3.4.5. Carbon Preference Index (CPI)

This is an indicator for dominance of natural hydrocarbons over the anthropogenic ones. It reveals the ratio of odd to even carbon number n-alkanes from different categories. Terrestrial vascular plants sourced hydrocarbons are identified with CPI values in the range of 3 to 10 while lower value close to 1 indicates petroleum and anthropogenic activities such as combustion of fossil fuel, effluent discharge, and agricultural and wood rubbles mostly from industrial and urban areas [9, 28, 84].

(1) $$\begin{array}{c} {\text{CPI}}_{25\text{–}33}=0.5*\left[\;\frac{\left(C_{25}\text{–}C_{33}\right)}{\left(C_{24}\text{–}C_{32}\right)}\;\right]+\left[\;\frac{\left(C_{25}\text{–}C_{33}\right)}{\left(C_{26}\text{–}C_{34}\right)}\;\right]. \end{array}$$

Table 5 shows CPI values obtained from this study in the range of 1 and 3, increasing towards the ocean. This shows that anthropogenic input decreases as marine input increases. Generally, the results indicate that Buffalo River Estuary is dominated by n-alkanes from both natural and anthropogenic origins [9, 84].

#### 3.4.6. C31/C19

This is another important ratio used to differentiate the sources of n-alkanes in water. The presence of nC31 is an indication of terrestrial biogenic hydrocarbons, whereas nC19 suggests marine biogenic inputs. The C31/C19 ratio is therefore used to assess the dominance of n-alkanes from either of the two sources. While ratio below 0.4 reveals marines sources, any value above 0.4 is an indication of land derived or nonmarine hydrocarbons [28, 78]. Although water samples from site E1 had no nC31, other sampling locations gave higher values than 0.4 (Tables 3 and 4), which are indicative of hydrocarbons mainly from anthropogenic origins.

#### 3.4.7. Average Carbon Length (ACL)

Average carbon length is an index used in the evaluation of odd carbon dominance per molecule in environmental samples to establish the link with higher plants normal alkanes. The value is usually constant in nonpolluted sites but fluctuates with depleted values in the areas polluted with petroleum hydrocarbons [85]. The values were calculated using the formula below as previously demonstrated [86, 87].

(2) ACL  value=25nC25+27nC27+29nC29+31nC31+33nC33C25+C27+C29+C31+C33.

**Figure 7:**
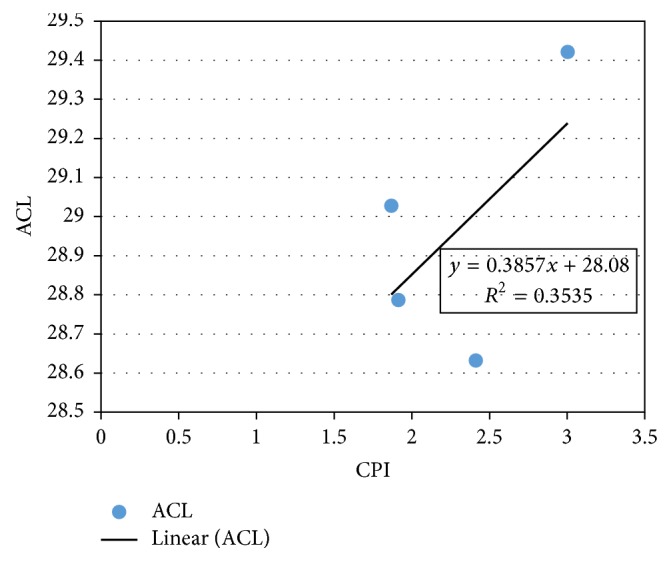
Plot of ACL versus CPI in the sediment compartment of Buffalo River Estuary.

ACL in the Buffalo River Estuary sediments ranged between 27.38 and 30.69 (Table 5). This shows about 3.31 units of changes. The little deviations from the mean value were noticed in January, February, and May. Three of the four stations were involved in the fluctuations, except E1 that had constant value close to the mean all through. The lowest ratio was obtained in May at E2 (27.38) and the highest in February at E4 (30.69). The slight fluctuations observed across the sampling stations indicate a little anthropogenic contribution to the abundance of hydrocarbons and corroborate previous reports elsewhere [9, 86]. The results show a positive significant correlation with CPI (*r* = 0.699; *p* < 0.01), though with a lower regression (*R*^2^ = 0.3535) (Figure 7), indicating a rise in ACL as CPI increases [29]. This relationship confirms that hydrocarbons in the Buffalo River Estuary are from both natural and anthropogenic origins because the CPI values were between 1 and 3 [78].

## 4. Conclusion

Physicochemical parameters of the Buffalo River Estuary water were assessed in this study. Almost all were found within the acceptable threshold limits except pH, turbidity, and total suspended solids that slightly exceeded, especially in May when the water was disturbed with a fresh and continual discharge of effluent at E3. Low dissolved oxygen concentration was equally observed in summer (February) at E2. In the same vein, the TPH concentrations in the estuary water and sediments ranged from 7.65 to 477 *μ*g/L and 12.59 to 1,100 mg/kg, respectively. Highest concentrations were observed principally at E1 (Buffalo River inflow) and E3 (second creek), and this possibly may be due to the industrial effluent discharge, leachate from a dumpsite close by, and urban/agricultural runoffs. Generally, the observed TPH concentration range signifies moderate to heavy pollution levels in the estuarine sediments.

To the best of our knowledge, this work on the petroleum hydrocarbon fingerprints of water and sediment in the estuary, first of its kind within the Buffalo City Metropolitan Municipality, has been able to provide baseline data on the current status of the estuary water. Diagnostic ratios used as biomarkers revealed that hydrocarbons in the area were from both natural and anthropogenic sources. Proper and continuous monitoring of the quality parameters should therefore be carried out so as to allow for effective pollution control in the aquatic environment.

## Figures and Tables

**Table 1:** Description of the Buffalo River Estuary.

Study area	Locations	Latitude	Longitude	Depth (m)	Description
Buffalo River Estuary	E1	33.0306°S	27.8580°E	2.40	A shallow entry point of the Buffalo River water into the estuary with pollution load from major towns like King Williams Town, Mdantsane, and Zwelisha.
	E2	33.0279°S	27.8630°E	3.47	An extension of the shallow part of the estuary, surrounded by hills with green vegetations.
	E3	33.0261°S	27.8830°E	4.44	Second creek: the largest perennial stream that accumulates stormwater, runoff, and leachates from Wilsonia industrial area, residential zones, central sewage treatment works, and a closed solid waste landfill site.
	E4	33.0243°S	27.8907°E	6.16	First creek: this is another stream under Steve Biko Bridge that contributes nonpoint source pollution in the form of sewerage, stormwater, vehicular emissions, and domestic and industrial runoff into the estuary. The point is very close to the Sea Spirit Fish Market that uses some fishing boats for its activities.
	E5	33.0233°S	27.8935°E	7.23	This is the deepest sampling point, located very close to Yacht club under Buffalo Bridge.

**Table 2:** Monthly variation in the physicochemical parameters of the estuary water.

Parameters	Summer		Autumn			Range	Total mean
	January	February	March	April	May		
pH	9.152	8.77	7.538	7.57	7.93	7.4–9.3	8.2 ± 0.7
Temperature (°C)	21.89	24.06	20.36	20.49	17.42	17–25	21 ± 2
Conductivity (*μ*S/m)	29.29	26.22	32.91	32.62	37.87	24.20–40.17	31.78 ± 4.80
Turbidity (NTU)	51.02	39.37	49.06	51.15	48.72	22.90–100.00	47.86 ± 19.58
Salinity (PSU)	26.29	24.21	27.04	26.34	27.74	22.84–29.10	26.32 ± 1.69
Dissolved oxygen (mg/L)	7.19	3.624	5.95	6.42	7.15	2.41–9.19	6.07 ± 1.62
Total suspended solids (mg/L)	9.93	10.77	11.23	11.50	92.13	1.33–446.67	27.11 ± 17.51
Total dissolved solids (mg/L)	18.89	16.11	20.9	21.06	19.04	13.50–22.75	19.20 ± 2.48

**Table 3:** Matrix of Pearson correlation among water quality parameters.

	pH	Temp	Cond	Turb	Sal	DO	TSS	TDS	TPH
pH	**1**								
Temp	**0.888** ^*∗∗*^	**1**							
Cond	**0.560** ^*∗∗*^	0.381	**1**						
Turb	0.325	0.317	0.201	**1**					
Sal	**0.861** ^*∗∗*^	**0.864** ^*∗∗*^	**0.670** ^*∗∗*^	0.245	**1**				
DO	−0.032	−0.316	**0.405** ^*∗*^	−0.183	0.047	**1**			
TSS	0.037	−0.110	0.162	**0.559** ^*∗∗*^	−0.076	0.020	**1**		
TDS	**0.451** ^*∗*^	**0.391** ^*∗*^	**0.835** ^*∗∗*^	0.129	**0.717** ^*∗∗*^	**0.406** ^*∗*^	−0.003	**1**	
TPH	0.046	−0.072	0.354	0.094	0.043	−0.004	0.333	−0.011	**1**

Temp: temperature; Cond: conductivity; Turb: turbidity; Sal: salinity; DO: dissolved oxygen; TSS: total suspended solids; TDS: total dissolved solids; TPH: total petroleum hydrocarbon. ^*∗∗*^Correlation is significant at the 0.01 level (2-tailed). ^*∗*^Correlation is significant at the 0.05 level (2-tailed).

**Table 4:** Average concentration of hydrocarbons and sources diagnostic ratios in the estuary water (*µ*g/L).

Parameters	Summer		Autumn			Range	Total mean
	January	February	March	April	May		
Total n-alkanes	77.63	161.37	68.29	53.47	177.34	15.46–328.59	105.38 ± 17.01
UCM	31.23	45.15	45.36	0	150.31	1.67–278.71	66.66 ± 16.78
TPH	102.61	174.24	104.58	53.47	297.59	7.65–477.07	146.50 ± 27.96
C15–C19 (odd)	0	0	16.61	0	16.97	0–44.33	6.72 ± 2.42
C18–C22 (even)	0	0.82	5.21	4.73	13.61	0–33.45	4.88 ± 1.69
C25–C35	71.21	108.38	2.77	6.54	88.17	0–228.93	55.41 ± 13.81
L/H	0	0.01	4.66	0.29	0.29	0–7.56	1.00 ± 0.40
C31/C19	0	0	0	0	5.53	0–16.98	2.46 ± 1.11
U/R	0.30	0.32	0.45	0	0.68	0–1.41	0.35 ± 0.09

**Table 5:** Average concentration of hydrocarbons and sources diagnostic ratios in the estuary sediment (mg/kg).

Parameters	Summer		Autumn			Range	Total mean
	January	February	March	April	May		
Total n-alkanes	55.35	38.45	25.48	89.02	35.95	2.04–169.89	49.53 ± 9.98
UCM	168.43	58.20	75.68	395.65	84.48	2.70–930.33	160.28 ± 54.19
TPH	223.78	96.65	101.16	484.68	120.43	12.59–1,100	209.81 ± 63.82
C15–C19 (odd)	1.08	0.44	0.92	2.88	0.71	0.25–6.30	1.23 ± 0.31
C18–C22 (even)	2.15	1.98	1.67	5.35	1.74	0.95–11.16	2.62 ± 0.54
C25–C35	43.53	29.26	18.33	63.60	19.33	6.43–127.12	35.62 ± 7.90
L/H	0.15	0.08	0.08	0.07	0.17	0.04–0.32	0.11 ± 0.02
C31/C19	14.50	26.38	3.51	3.80	5.08	2.30–35.29	10.95 ± 2.33
U/R	1.53	1.54	3.19	3.82	2.19	0.27–5.77	2.47 ± 0.38
CPI	3.00	2.99	1.94	1.68	1.52	1.14–4.11	2.26 ± 0.20
ACL	29.37	29.37	28.42	28.92	28.53	27.38–30.69	28.94 ± 0.16

UCM: unresolved complex mixture; TPH: total petroleum hydrocarbon; L/H: low molecular n-alkanes/high molecular n-alkanes; U/R: unresolved n-alkanes/resolved n-alkanes; CPI: carbon preference index; ACL: average carbon chain length.

**Table 6:** Percentage moisture, organic carbon, and organic matter contents of the sediments.

	Moisture (%)	Organic carbon (%)	Organic matter (%)
Range	45.01–67.86	3.12–8.94	5.39–15.42
Mean	59.03 ± 6.85	6.14 ± 1.35	10.59 ± 2.32

**Table 7:** Pearson correlations among the sediments quality parameters.

	% moisture	% OC	% OM	TPH
% moisture	1			
% OC	0.316	1		
% OM	0.316	**1.000** ^*∗∗*^	1	
TPH	0.133	**0.665** ^*∗∗*^	**0.665** ^*∗∗*^	1

^*∗∗*^Correlation is significant at the 0.01 level (2-tailed).
